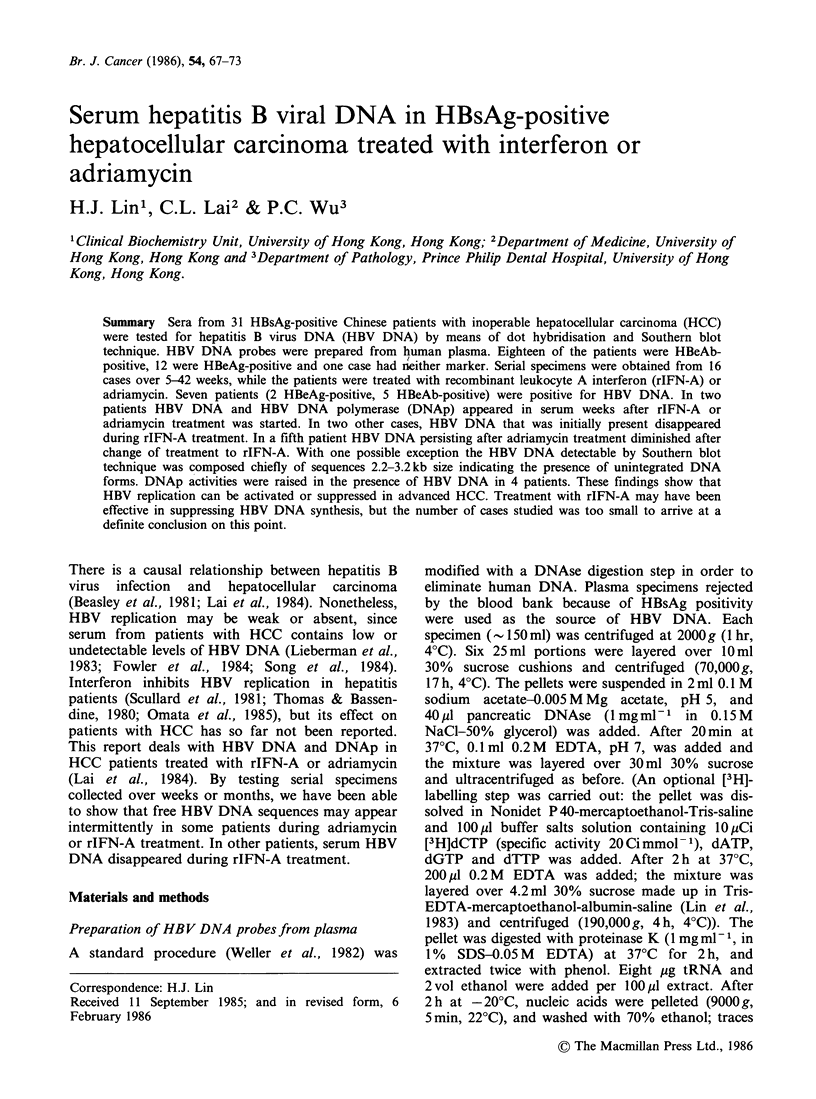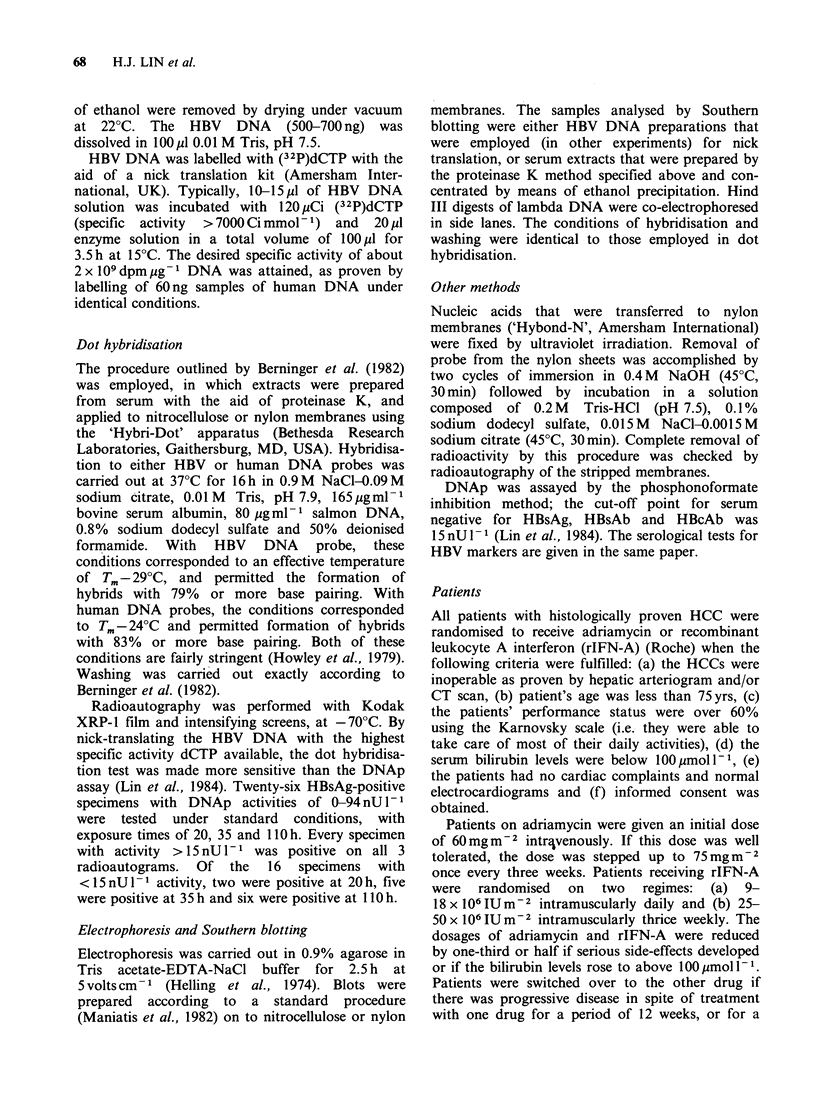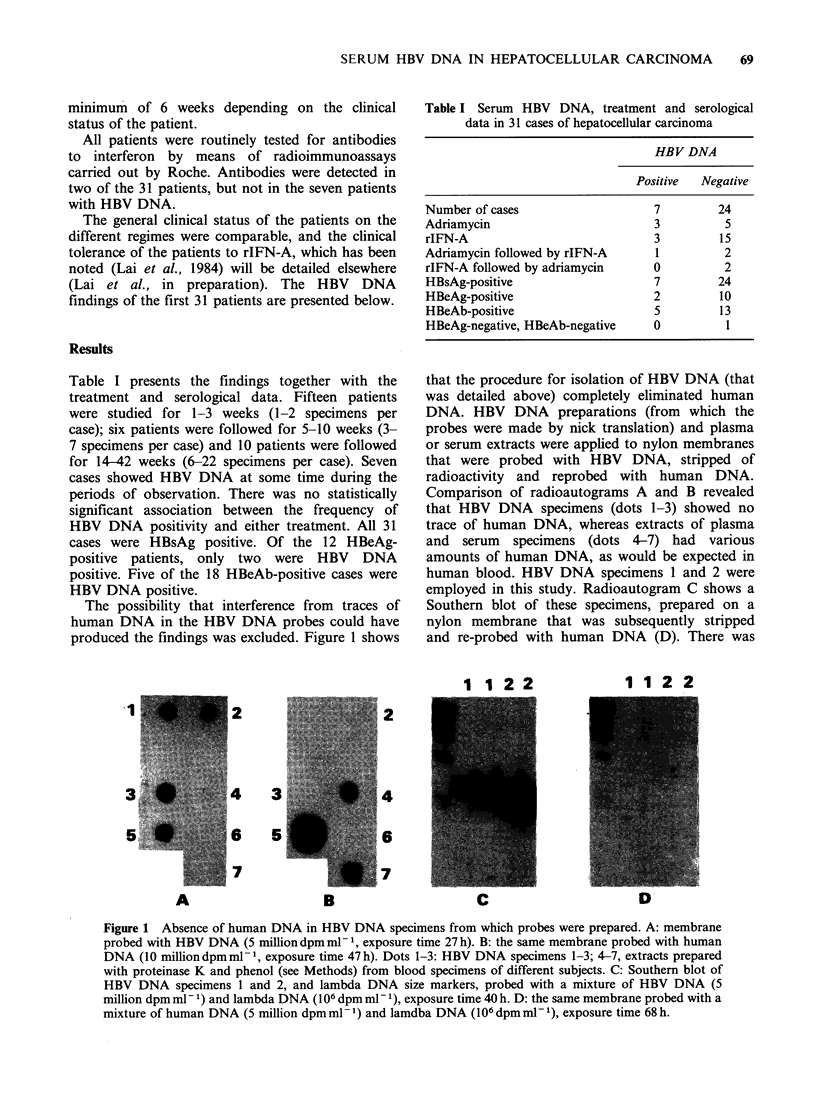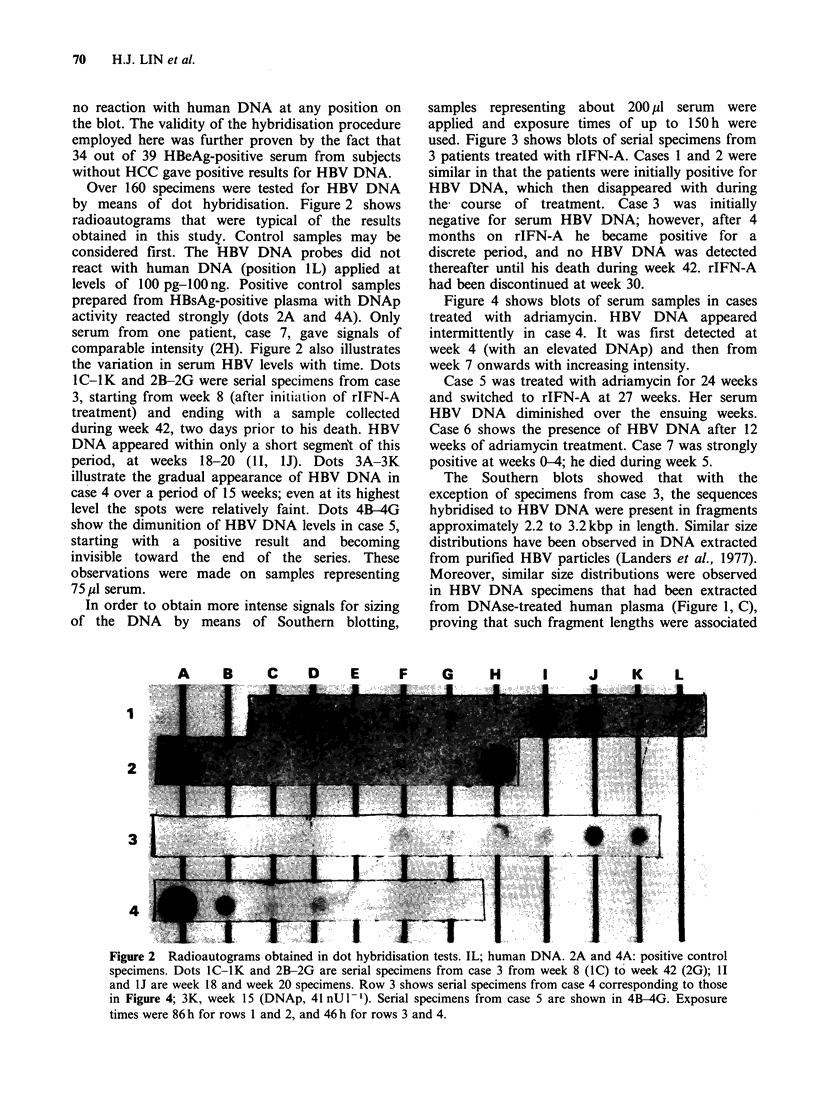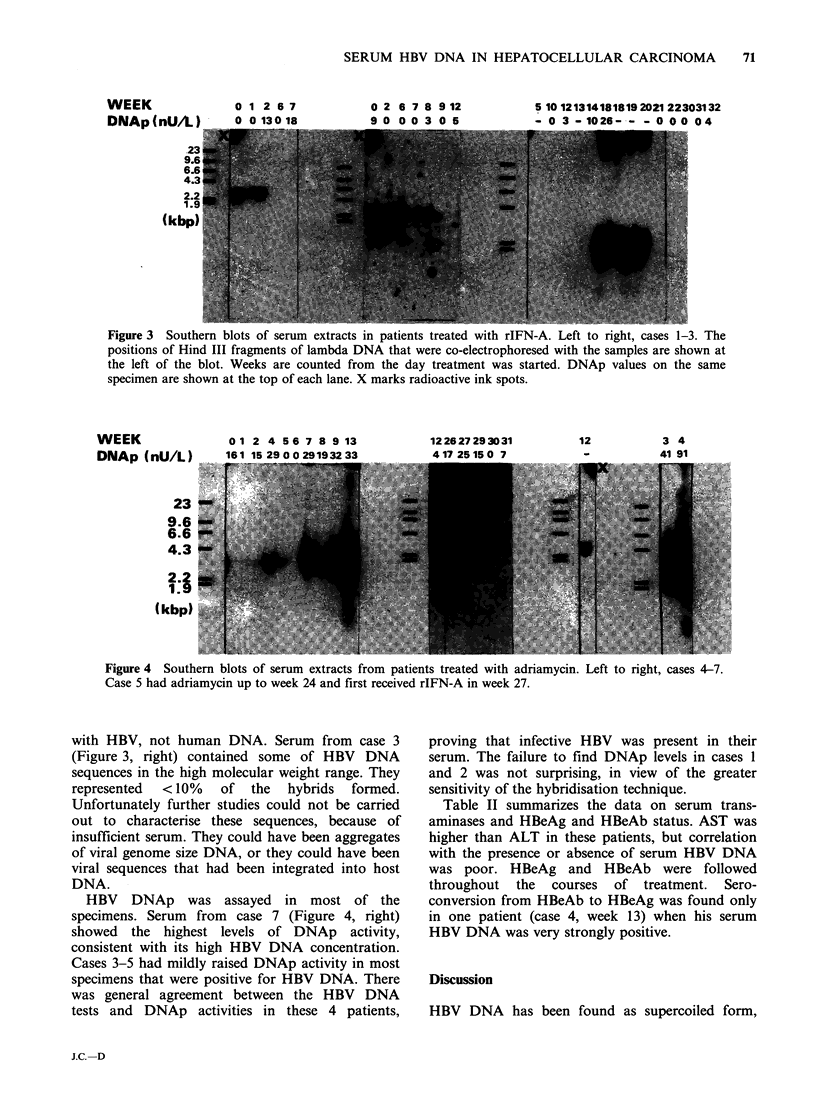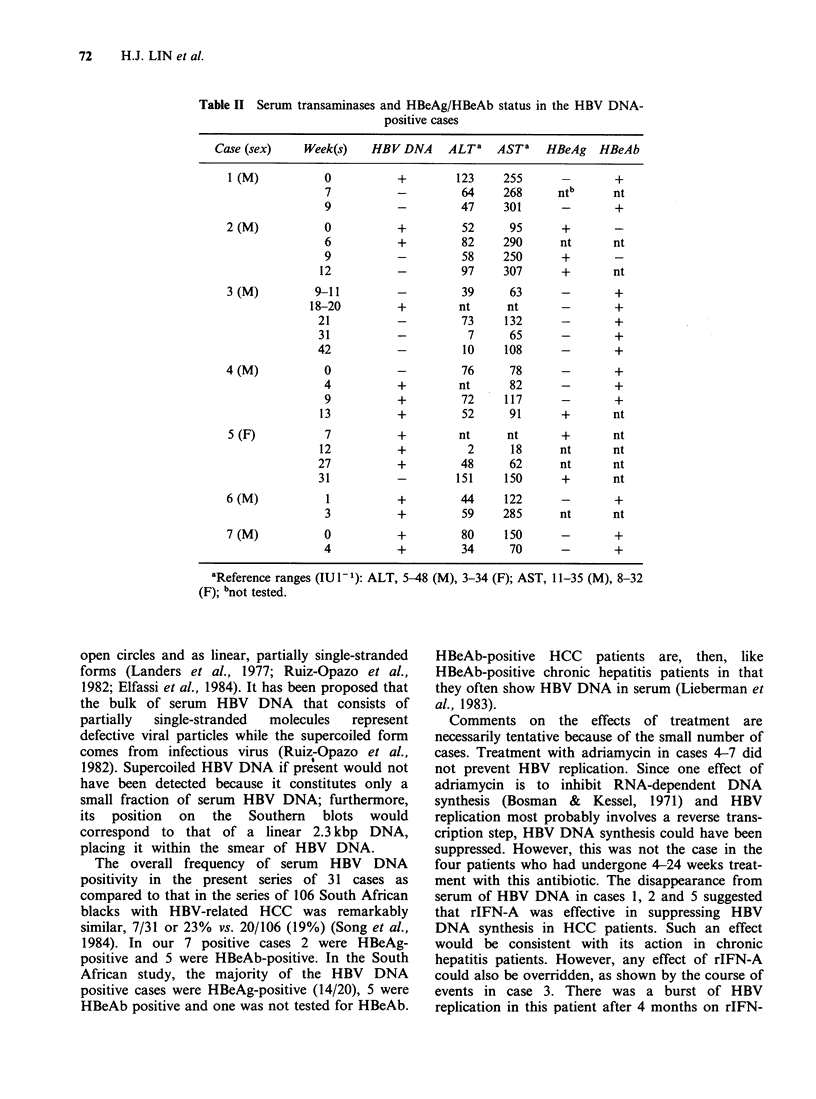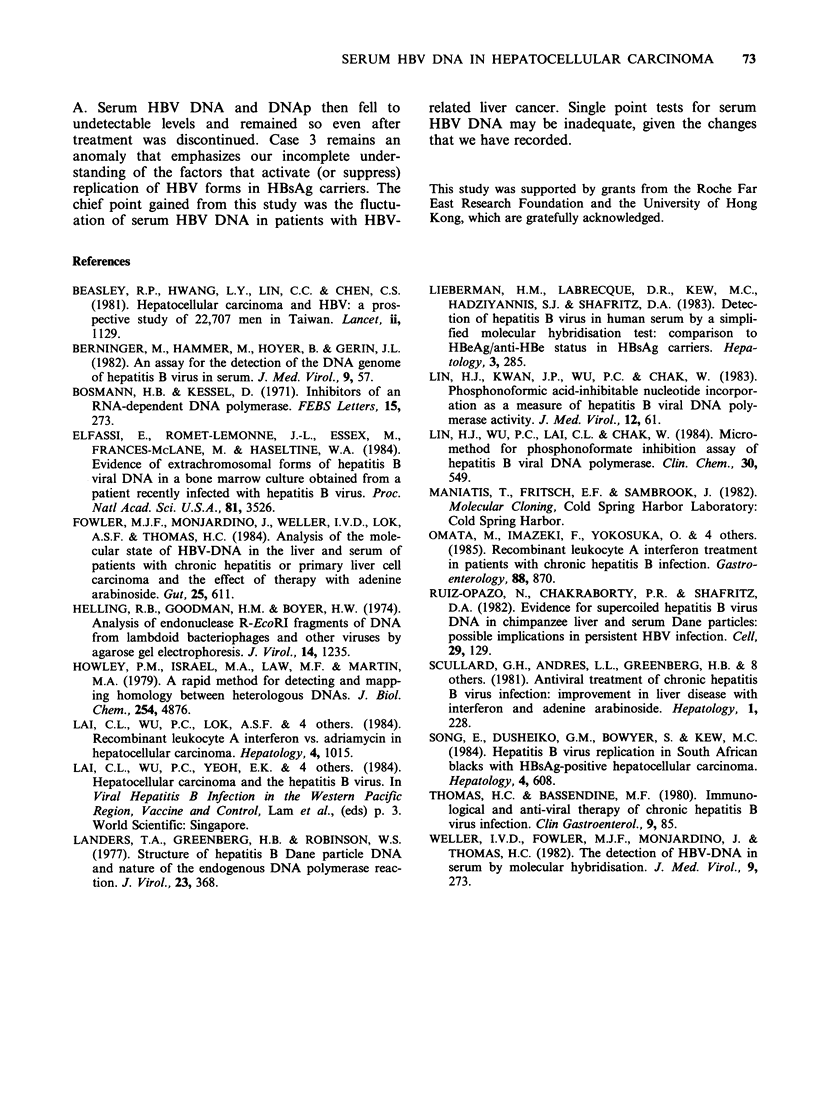# Serum hepatitis B viral DNA in HBsAg-positive hepatocellular carcinoma treated with interferon or adriamycin.

**DOI:** 10.1038/bjc.1986.153

**Published:** 1986-07

**Authors:** H. J. Lin, C. L. Lai, P. C. Wu

## Abstract

**Images:**


*Br. J. Cancer* (1986), **54**, 67–73

### Serum hepatitis B viral DNA in HBsAg-positive hepatocellular carcinoma treated with interferon or adriamycin

H.J. Lin[1], C.L. Lai[2] & P.C. Wu[3]

[1]*Clinical Biochemistry Unit, University of Hong Kong, Hong Kong;* [2]*Department of Medicine, University of Hong Kong, Hong Kong and* [3]*Department of Pathology, Prince Philip Dental Hospital, University of Hong Kong, Hong Kong.*

**Summary** Sera from 31 HBsAg-positive Chinese patients with inoperable hepatocellular carcinoma (HCC) were tested for hepatitis B virus DNA (HBV DNA) by means of dot hybridisation and Southern blot technique. HBV DNA probes were prepared from human plasma. Eighteen of the patients were HBeAb-positive, 12 were HBeAg-positive and one case had neither marker. Serial specimens were obtained from 16 cases over 5–42 weeks, while the patients were treated with recombinant leukocyte A interferon (rIFN-A) or adriamycin. Seven patients (2 HBeAg-positive, 5 HBeAb-positive) were positive for HBV DNA. In two patients HBV DNA and HBV DNA polymerase (DNAp) appeared in serum weeks after rIFN-A or adriamycin treatment was started. In two other cases, HBV DNA that was initially present disappeared during rIFN-A treatment. In a fifth patient HBV DNA persisting after adriamycin treatment diminished after change of treatment to rIFN-A. With one possible exception the HBV DNA detectable by Southern blot technique was composed chiefly of sequences 2.2–3.2 kb size indicating the presence of unintegrated DNA forms. DNAp activities were raised in the presence of HBV DNA in 4 patients. These findings show that HBV replication can be activated or suppressed in advanced HCC. Treatment with rIFN-A may have been effective in suppressing HBV DNA synthesis, but the number of cases studied was too small to arrive at a definite conclusion on this point.

There is a causal relationship between hepatitis B virus infection and hepatocellular carcinoma (Beasley *et al.*, 1981; Lai *et al.*, 1984). Nonetheless, HBV replication may be weak or absent, since serum from patients with HCC contains low or undetectable levels of HBV DNA (Lieberman *et al.*, 1983; Fowler *et al.*, 1984; Song *et al.*, 1984). Interferon inhibits HBV replication in hepatitis patients (Scullard *et al.*, 1981; Thomas & Bassendine, 1980; Omata *et al.*, 1985), but its effect on patients with HCC has so far not been reported. This report deals with HBV DNA and DNAp in HCC patients treated with rIFN-A or adriamycin (Lai *et al.*, 1984). By testing serial specimens collected over weeks or months, we have been able to show that free HBV DNA sequences may appear intermittently in some patients during adriamycin or rIFN-A treatment. In other patients, serum HBV DNA disappeared during rIFN-A treatment.

### Materials and methods

*Preparation of HBV DNA probes from plasma*

A standard procedure (Weller *et al.*, 1982) was modified with a DNAse digestion step in order to eliminate human DNA. Plasma specimens rejected by the blood bank because of HBsAg positivity were used as the source of HBV DNA. Each specimen (~150 ml) was centrifuged at 2000 *g* (1 hr, 4°C). Six 25 ml portions were layered over 10 ml 30% sucrose cushions and centrifuged (70,000 *g*, 17 h, 4°C). The pellets were suspended in 2 ml 0.1 M sodium acetate–0.005 M Mg acetate, pH 5, and 40 $\mu$l pancreatic DNAse (1 mg ml$^{-1}$ in 0.15 M NaCl–50% glycerol) was added. After 20 min at 37°C, 0.1 ml 0.2 M EDTA, pH 7, was added and the mixture was layered over 30 ml 30% sucrose and ultracentrifuged as before. (An optional [$^3$H]-labelling step was carried out: the pellet was dissolved in Nonidet P 40-mercaptoethanol-Tris-saline and 100 $\mu$l buffer salts solution containing 10 $\mu$Ci [$^3$H]dCTP (specific activity 20 Ci mmol$^{-1}$), dATP, dGTP and dTTP was added. After 2 h at 37°C, 200 $\mu$l 0.2 M EDTA was added; the mixture was layered over 4.2 ml 30% sucrose made up in Tris-EDTA-mercaptoethanol-albumin-saline (Lin *et al.*, 1983) and centrifuged (190,000 *g*, 4 h, 4°C)). The pellet was digested with proteinase K (1 mg ml$^{-1}$, in 1% SDS–0.05 M EDTA) at 37°C for 2 h, and extracted twice with phenol. Eight $\mu$g tRNA and 2 vol ethanol were added per 100 $\mu$l extract. After 2 h at −20°C, nucleic acids were pelleted (9000 *g*, 5 min, 22°C), and washed with 70% ethanol; traces

Correspondence: H.J. Lin

Received 11 September 1985; and in revised form, 6 February 1986

© The Macmillan Press Ltd., 1986

of ethanol were removed by drying under vacuum at 22°C. The HBV DNA (500–700 ng) was dissolved in 100 $\mu$l 0.01 M Tris, pH 7.5.

HBV DNA was labelled with ($^{32}$P)dCTP with the aid of a nick translation kit (Amersham International, UK). Typically, 10–15 $\mu$l of HBV DNA solution was incubated with 120 $\mu$Ci ($^{32}$P)dCTP (specific activity >7000 Ci mmol$^{-1}$) and 20 $\mu$l enzyme solution in a total volume of 100 $\mu$l for 3.5 h at 15°C. The desired specific activity of about $2 \times 10^9$ dpm $\mu$g$^{-1}$ DNA was attained, as proven by labelling of 60 ng samples of human DNA under identical conditions.

### *Dot hybridisation*

The procedure outlined by Berninger *et al.* (1982) was employed, in which extracts were prepared from serum with the aid of proteinase K, and applied to nitrocellulose or nylon membranes using the 'Hybri-Dot' apparatus (Bethesda Research Laboratories, Gaithersburg, MD, USA). Hybridisation to either HBV or human DNA probes was carried out at 37°C for 16 h in 0.9 M NaCl–0.09 M sodium citrate, 0.01 M Tris, pH 7.9, 165 $\mu$g ml$^{-1}$ bovine serum albumin, 80 $\mu$g ml$^{-1}$ salmon DNA, 0.8% sodium dodecyl sulfate and 50% deionised formamide. With HBV DNA probe, these conditions corresponded to an effective temperature of $T_m - 29$°C, and permitted the formation of hybrids with 79% or more base pairing. With human DNA probes, the conditions corresponded to $T_m - 24$°C and permitted formation of hybrids with 83% or more base pairing. Both of these conditions are fairly stringent (Howley *et al.*, 1979). Washing was carried out exactly according to Berninger *et al.* (1982).

Radioautography was performed with Kodak XRP-1 film and intensifying screens, at −70°C. By nick-translating the HBV DNA with the highest specific activity dCTP available, the dot hybridisation test was made more sensitive than the DNAp assay (Lin *et al.*, 1984). Twenty-six HBsAg-positive specimens with DNAp activities of 0–94 nU l$^{-1}$ were tested under standard conditions, with exposure times of 20, 35 and 110 h. Every specimen with activity >15 nU l$^{-1}$ was positive on all 3 radioautograms. Of the 16 specimens with <15 nU l$^{-1}$ activity, two were positive at 20 h, five were positive at 35 h and six were positive at 110 h.

### *Electrophoresis and Southern blotting*

Electrophoresis was carried out in 0.9% agarose in Tris acetate-EDTA-NaCl buffer for 2.5 h at 5 volts cm$^{-1}$ (Helling *et al.*, 1974). Blots were prepared according to a standard procedure (Maniatis *et al.*, 1982) on to nitrocellulose or nylon membranes. The samples analysed by Southern blotting were either HBV DNA preparations that were employed (in other experiments) for nick translation, or serum extracts that were prepared by the proteinase K method specified above and concentrated by means of ethanol precipitation. Hind III digests of lambda DNA were co-electrophoresed in side lanes. The conditions of hybridisation and washing were identical to those employed in dot hybridisation.

### *Other methods*

Nucleic acids that were transferred to nylon membranes ('Hybond-N', Amersham International) were fixed by ultraviolet irradiation. Removal of probe from the nylon sheets was accomplished by two cycles of immersion in 0.4 M NaOH (45°C, 30 min) followed by incubation in a solution composed of 0.2 M Tris-HCl (pH 7.5), 0.1% sodium dodecyl sulfate, 0.015 M NaCl–0.0015 M sodium citrate (45°C, 30 min). Complete removal of radioactivity by this procedure was checked by radioautography of the stripped membranes.

DNAp was assayed by the phosphonoformate inhibition method; the cut-off point for serum negative for HBsAg, HBsAb and HBcAb was 15 nU l$^{-1}$ (Lin *et al.*, 1984). The serological tests for HBV markers are given in the same paper.

### *Patients*

All patients with histologically proven HCC were randomised to receive adriamycin or recombinant leukocyte A interferon (rIFN-A) (Roche) when the following criteria were fulfilled: (a) the HCCs were inoperable as proven by hepatic arteriogram and/or CT scan, (b) patient's age was less than 75 yrs, (c) the patients' performance status were over 60% using the Karnovsky scale (i.e. they were able to take care of most of their daily activities), (d) the serum bilirubin levels were below 100 $\mu$mol l$^{-1}$, (e) the patients had no cardiac complaints and normal electrocardiograms and (f) informed consent was obtained.

Patients on adriamycin were given an initial dose of 60 mg m$^{-2}$ intravenously. If this dose was well tolerated, the dose was stepped up to 75 mg m$^{-2}$ once every three weeks. Patients receiving rIFN-A were randomised on two regimes: (a) 9–$18 \times 10^6$ IU m$^{-2}$ intramuscularly daily and (b) 25–$50 \times 10^6$ IU m$^{-2}$ intramuscularly thrice weekly. The dosages of adriamycin and rIFN-A were reduced by one-third or half if serious side-effects developed or if the bilirubin levels rose to above 100 $\mu$mol l$^{-1}$. Patients were switched over to the other drug if there was progressive disease in spite of treatment with one drug for a period of 12 weeks, or for a minimum of 6 weeks depending on the clinical status of the patient.

All patients were routinely tested for antibodies to interferon by means of radioimmunoassays carried out by Roche. Antibodies were detected in two of the 31 patients, but not in the seven patients with HBV DNA.

The general clinical status of the patients on the different regimes were comparable, and the clinical tolerance of the patients to rIFN-A, which has been noted (Lai *et al.*, 1984) will be detailed elsewhere (Lai *et al.*, in preparation). The HBV DNA findings of the first 31 patients are presented below.

**Table I** Serum HBV DNA, treatment and serological data in 31 cases of hepatocellular carcinoma

| | *HBV DNA* | |
|---|---|---|
| | *Positive* | *Negative* |
| Number of cases | 7 | 24 |
| Adriamycin | 3 | 5 |
| rIFN-A | 3 | 15 |
| Adriamycin followed by rIFN-A | 1 | 2 |
| rIFN-A followed by adriamycin | 0 | 2 |
| HBsAg-positive | 7 | 24 |
| HBeAg-positive | 2 | 10 |
| HBeAb-positive | 5 | 13 |
| HBeAg-negative, HBeAb-negative | 0 | 1 |

### Results

Table I presents the findings together with the treatment and serological data. Fifteen patients were studied for 1–3 weeks (1–2 specimens per case); six patients were followed for 5–10 weeks (3–7 specimens per case) and 10 patients were followed for 14–42 weeks (6–22 specimens per case). Seven cases showed HBV DNA at some time during the periods of observation. There was no statistically significant association between the frequency of HBV DNA positivity and either treatment. All 31 cases were HBsAg positive. Of the 12 HBeAg-positive patients, only two were HBV DNA positive. Five of the 18 HBeAb-positive cases were HBV DNA positive.

The possibility that interference from traces of human DNA in the HBV DNA probes could have produced the findings was excluded. Figure 1 shows that the procedure for isolation of HBV DNA (that was detailed above) completely eliminated human DNA. HBV DNA preparations (from which the probes were made by nick translation) and plasma or serum extracts were applied to nylon membranes that were probed with HBV DNA, stripped of radioactivity and reprobed with human DNA. Comparison of radioautograms A and B revealed that HBV DNA specimens (dots 1–3) showed no trace of human DNA, whereas extracts of plasma and serum specimens (dots 4–7) had various amounts of human DNA, as would be expected in human blood. HBV DNA specimens 1 and 2 were employed in this study. Radioautogram C shows a Southern blot of these specimens, prepared on a nylon membrane that was subsequently stripped and re-probed with human DNA (D). There was

**Figure 1** Absence of human DNA in HBV DNA specimens from which probes were prepared. A: membrane probed with HBV DNA (5 million dpm $ml^{-1}$, exposure time 27 h). B: the same membrane probed with human DNA (10 million dpm $ml^{-1}$, exposure time 47 h). Dots 1–3: HBV DNA specimens 1–3; 4–7, extracts prepared with proteinase K and phenol (see Methods) from blood specimens of different subjects. C: Southern blot of HBV DNA specimens 1 and 2, and lambda DNA size markers, probed with a mixture of HBV DNA (5 million dpm $ml^{-1}$) and lambda DNA ($10^6$ dpm $ml^{-1}$), exposure time 40 h. D: the same membrane probed with a mixture of human DNA (5 million dpm $ml^{-1}$) and lamdba DNA ($10^6$ dpm $ml^{-1}$), exposure time 68 h.

no reaction with human DNA at any position on the blot. The validity of the hybridisation procedure employed here was further proven by the fact that 34 out of 39 HBeAg-positive serum from subjects without HCC gave positive results for HBV DNA.

Over 160 specimens were tested for HBV DNA by means of dot hybridisation. Figure 2 shows radioautograms that were typical of the results obtained in this study. Control samples may be considered first. The HBV DNA probes did not react with human DNA (position 1L) applied at levels of 100 pg–100 ng. Positive control samples prepared from HBsAg-positive plasma with DNAp activity reacted strongly (dots 2A and 4A). Only serum from one patient, case 7, gave signals of comparable intensity (2H). Figure 2 also illustrates the variation in serum HBV levels with time. Dots 1C–1K and 2B–2G were serial specimens from case 3, starting from week 8 (after initiation of rIFN-A treatment) and ending with a sample collected during week 42, two days prior to his death. HBV DNA appeared within only a short segment of this period, at weeks 18–20 (1I, 1J). Dots 3A–3K illustrate the gradual appearance of HBV DNA in case 4 over a period of 15 weeks; even at its highest level the spots were relatively faint. Dots 4B–4G show the dimunition of HBV DNA levels in case 5, starting with a positive result and becoming invisible toward the end of the series. These observations were made on samples representing 75 $\mu$l serum.

In order to obtain more intense signals for sizing of the DNA by means of Southern blotting, samples representing about 200 $\mu$l serum were applied and exposure times of up to 150 h were used. Figure 3 shows blots of serial specimens from 3 patients treated with rIFN-A. Cases 1 and 2 were similar in that the patients were initially positive for HBV DNA, which then disappeared with during the course of treatment. Case 3 was initially negative for serum HBV DNA; however, after 4 months on rIFN-A he became positive for a discrete period, and no HBV DNA was detected thereafter until his death during week 42. rIFN-A had been discontinued at week 30.

Figure 4 shows blots of serum samples in cases treated with adriamycin. HBV DNA appeared intermittently in case 4. It was first detected at week 4 (with an elevated DNAp) and then from week 7 onwards with increasing intensity.

Case 5 was treated with adriamycin for 24 weeks and switched to rIFN-A at 27 weeks. Her serum HBV DNA diminished over the ensuing weeks. Case 6 shows the presence of HBV DNA after 12 weeks of adriamycin treatment. Case 7 was strongly positive at weeks 0–4; he died during week 5.

The Southern blots showed that with the exception of specimens from case 3, the sequences hybridised to HBV DNA were present in fragments approximately 2.2 to 3.2 kbp in length. Similar size distributions have been observed in DNA extracted from purified HBV particles (Landers *et al.*, 1977). Moreover, similar size distributions were observed in HBV DNA specimens that had been extracted from DNAse-treated human plasma (Figure 1, C), proving that such fragment lengths were associated

**Figure 2** Radioautograms obtained in dot hybridisation tests. IL; human DNA. 2A and 4A: positive control specimens. Dots 1C–1K and 2B–2G are serial specimens from case 3 from week 8 (1C) to week 42 (2G); 1I and 1J are week 18 and week 20 specimens. Row 3 shows serial specimens from case 4 corresponding to those in **Figure 4**; 3K, week 15 (DNAp, 41 nU l$^{-1}$). Serial specimens from case 5 are shown in 4B–4G. Exposure times were 86 h for rows 1 and 2, and 46 h for rows 3 and 4.

**Figure 3** Southern blots of serum extracts in patients treated with rIFN-A. Left to right, cases 1–3. The positions of Hind III fragments of lambda DNA that were co-electrophoresed with the samples are shown at the left of the blot. Weeks are counted from the day treatment was started. DNAp values on the same specimen are shown at the top of each lane. X marks radioactive ink spots.

**Figure 4** Southern blots of serum extracts from patients treated with adriamycin. Left to right, cases 4–7. Case 5 had adriamycin up to week 24 and first received rIFN-A in week 27.

with HBV, not human DNA. Serum from case 3 (Figure 3, right) contained some of HBV DNA sequences in the high molecular weight range. They represented <10% of the hybrids formed. Unfortunately further studies could not be carried out to characterise these sequences, because of insufficient serum. They could have been aggregates of viral genome size DNA, or they could have been viral sequences that had been integrated into host DNA.

HBV DNAp was assayed in most of the specimens. Serum from case 7 (Figure 4, right) showed the highest levels of DNAp activity, consistent with its high HBV DNA concentration. Cases 3–5 had mildly raised DNAp activity in most specimens that were positive for HBV DNA. There was general agreement between the HBV DNA tests and DNAp activities in these 4 patients, proving that infective HBV was present in their serum. The failure to find DNAp levels in cases 1 and 2 was not surprising, in view of the greater sensitivity of the hybridisation technique.

Table II summarizes the data on serum transaminases and HBeAg and HBeAb status. AST was higher than ALT in these patients, but correlation with the presence or absence of serum HBV DNA was poor. HBeAg and HBeAb were followed throughout the courses of treatment. Seroconversion from HBeAb to HBeAg was found only in one patient (case 4, week 13) when his serum HBV DNA was very strongly positive.

### Discussion

HBV DNA has been found as supercoiled form,

**Table II** Serum transaminases and HBeAg/HBeAb status in the HBV DNA-positive cases

| *Case* (*sex*) | *Week*(*s*) | *HBV DNA* | *ALT*[a] | *AST*[a] | *HBeAg* | *HBeAb* |
|---|---|---|---|---|---|---|
| 1 (M) | 0 | + | 123 | 255 | – | + |
| | 7 | – | 64 | 268 | nt[b] | nt |
| | 9 | – | 47 | 301 | – | + |
| 2 (M) | 0 | + | 52 | 95 | + | – |
| | 6 | + | 82 | 290 | nt | nt |
| | 9 | – | 58 | 250 | + | – |
| | 12 | – | 97 | 307 | + | nt |
| 3 (M) | 9–11 | – | 39 | 63 | – | + |
| | 18–20 | + | nt | nt | – | + |
| | 21 | – | 73 | 132 | – | + |
| | 31 | – | 7 | 65 | – | + |
| | 42 | – | 10 | 108 | – | + |
| 4 (M) | 0 | – | 76 | 78 | – | + |
| | 4 | + | nt | 82 | – | + |
| | 9 | + | 72 | 117 | – | + |
| | 13 | + | 52 | 91 | + | nt |
| 5 (F) | 7 | + | nt | nt | + | nt |
| | 12 | + | 2 | 18 | nt | nt |
| | 27 | + | 48 | 62 | nt | nt |
| | 31 | – | 151 | 150 | + | nt |
| 6 (M) | 1 | + | 44 | 122 | – | + |
| | 3 | + | 59 | 285 | nt | nt |
| 7 (M) | 0 | + | 80 | 150 | – | + |
| | 4 | + | 34 | 70 | – | + |

[a]Reference ranges ($IU\,l^{-1}$): ALT, 5–48 (M), 3–34 (F); AST, 11–35 (M), 8–32 (F); [b]not tested.

open circles and as linear, partially single-stranded forms (Landers *et al.*, 1977; Ruiz-Opazo *et al.*, 1982; Elfassi *et al.*, 1984). It has been proposed that the bulk of serum HBV DNA that consists of partially single-stranded molecules represent defective viral particles while the supercoiled form comes from infectious virus (Ruiz-Opazo *et al.*, 1982). Supercoiled HBV DNA if present would not have been detected because it constitutes only a small fraction of serum HBV DNA; furthermore, its position on the Southern blots would correspond to that of a linear 2.3 kbp DNA, placing it within the smear of HBV DNA.

The overall frequency of serum HBV DNA positivity in the present series of 31 cases as compared to that in the series of 106 South African blacks with HBV-related HCC was remarkably similar, 7/31 or 23% *vs.* 20/106 (19%) (Song *et al.*, 1984). In our 7 positive cases 2 were HBeAg-positive and 5 were HBeAb-positive. In the South African study, the majority of the HBV DNA positive cases were HBeAg-positive (14/20), 5 were HBeAb positive and one was not tested for HBeAb. HBeAb-positive HCC patients are, then, like HBeAb-positive chronic hepatitis patients in that they often show HBV DNA in serum (Lieberman *et al.*, 1983).

Comments on the effects of treatment are necessarily tentative because of the small number of cases. Treatment with adriamycin in cases 4–7 did not prevent HBV replication. Since one effect of adriamycin is to inhibit RNA-dependent DNA synthesis (Bosman & Kessel, 1971) and HBV replication most probably involves a reverse transcription step, HBV DNA synthesis could have been suppressed. However, this was not the case in the four patients who had undergone 4–24 weeks treatment with this antibiotic. The disappearance from serum of HBV DNA in cases 1, 2 and 5 suggested that rIFN-A was effective in suppressing HBV DNA synthesis in HCC patients. Such an effect would be consistent with its action in chronic hepatitis patients. However, any effect of rIFN-A could also be overridden, as shown by the course of events in case 3. There was a burst of HBV replication in this patient after 4 months on rIFN-

A. Serum HBV DNA and DNAp then fell to undetectable levels and remained so even after treatment was discontinued. Case 3 remains an anomaly that emphasizes our incomplete understanding of the factors that activate (or suppress) replication of HBV forms in HBsAg carriers. The chief point gained from this study was the fluctuation of serum HBV DNA in patients with HBV-related liver cancer. Single point tests for serum HBV DNA may be inadequate, given the changes that we have recorded.

This study was supported by grants from the Roche Far East Research Foundation and the University of Hong Kong, which are gratefully acknowledged.

**References**

BEASLEY, R.P., HWANG, L.Y., LIN, C.C. & CHEN, C.S. (1981). Hepatocellular carcinoma and HBV: a prospective study of 22,707 men in Taiwan. *Lancet*, **ii**, 1129.

BERNINGER, M., HAMMER, M., HOYER, B. & GERIN, J.L. (1982). An assay for the detection of the DNA genome of hepatitis B virus in serum. *J. Med. Virol.*, **9**, 57.

BOSMANN, H.B. & KESSEL, D. (1971). Inhibitors of an RNA-dependent DNA polymerase. *FEBS Letters*, **15**, 273.

ELFASSI, E., ROMET-LEMONNE, J.-L., ESSEX, M., FRANCES-McLANE, M. & HASELTINE, W.A. (1984). Evidence of extrachromosomal forms of hepatitis B viral DNA in a bone marrow culture obtained from a patient recently infected with hepatitis B virus. *Proc. Natl Acad. Sci. U.S.A.*, **81**, 3526.

FOWLER, M.J.F., MONJARDINO, J., WELLER, I.V.D., LOK, A.S.F. & THOMAS, H.C. (1984). Analysis of the molecular state of HBV-DNA in the liver and serum of patients with chronic hepatitis or primary liver cell carcinoma and the effect of therapy with adenine arabinoside. *Gut*, **25**, 611.

HELLING, R.B., GOODMAN, H.M. & BOYER, H.W. (1974). Analysis of endonuclease R-*Eco*RI fragments of DNA from lambdoid bacteriophages and other viruses by agarose gel electrophoresis. *J. Virol.*, **14**, 1235.

HOWLEY, P.M., ISRAEL, M.A., LAW, M.F. & MARTIN, M.A. (1979). A rapid method for detecting and mapping homology between heterologous DNAs. *J. Biol. Chem.*, **254**, 4876.

LAI, C.L., WU, P.C., LOK, A.S.F. & 4 others. (1984). Recombinant leukocyte A interferon vs. adriamycin in hepatocellular carcinoma. *Hepatology*, **4**, 1015.

LAI, C.L., WU, P.C., YEOH, E.K. & 4 others. (1984). Hepatocellular carcinoma and the hepatitis B virus. In *Viral Hepatitis B Infection in the Western Pacific Region, Vaccine and Control*, Lam *et al.*, (eds) p. 3. World Scientific: Singapore.

LANDERS, T.A., GREENBERG, H.B. & ROBINSON, W.S. (1977). Structure of hepatitis B Dane particle DNA and nature of the endogenous DNA polymerase reaction. *J. Virol.*, **23**, 368.

LIEBERMAN, H.M., LABRECQUE, D.R., KEW, M.C., HADZIYANNIS, S.J. & SHAFRITZ, D.A. (1983). Detection of hepatitis B virus in human serum by a simplified molecular hybridisation test: comparison to HBeAg/anti-HBe status in HBsAg carriers. *Hepatology*, **3**, 285.

LIN, H.J., KWAN, J.P., WU, P.C. & CHAK, W. (1983). Phosphonoformic acid-inhibitable nucleotide incorporation as a measure of hepatitis B viral DNA polymerase activity. *J. Med. Virol.*, **12**, 61.

LIN, H.J., WU, P.C., LAI, C.L. & CHAK, W. (1984). Micromethod for phosphonoformate inhibition assay of hepatitis B viral DNA polymerase. *Clin. Chem.*, **30**, 549.

MANIATIS, T., FRITSCH, E.F. & SAMBROOK, J. (1982). *Molecular Cloning*, Cold Spring Harbor Laboratory: Cold Spring Harbor.

OMATA, M., IMAZEKI, F., YOKOSUKA, O. & 4 others. (1985). Recombinant leukocyte A interferon treatment in patients with chronic hepatitis B infection. *Gastroenterology*, **88**, 870.

RUIZ-OPAZO, N., CHAKRABORTY, P.R. & SHAFRITZ, D.A. (1982). Evidence for supercoiled hepatitis B virus DNA in chimpanzee liver and serum Dane particles: possible implications in persistent HBV infection. *Cell*, **29**, 129.

SCULLARD, G.H., ANDRES, L.L., GREENBERG, H.B. & 8 others. (1981). Antiviral treatment of chronic hepatitis B virus infection: improvement in liver disease with interferon and adenine arabinoside. *Hepatology*, **1**, 228.

SONG, E., DUSHEIKO, G.M., BOWYER, S. & KEW, M.C. (1984). Hepatitis B virus replication in South African blacks with HBsAg-positive hepatocellular carcinoma. *Hepatology*, **4**, 608.

THOMAS, H.C. & BASSENDINE, M.F. (1980). Immunological and anti-viral therapy of chronic hepatitis B virus infection. *Clin Gastroenterol.*, **9**, 85.

WELLER, I.V.D., FOWLER, M.J.F., MONJARDINO, J. & THOMAS, H.C. (1982). The detection of HBV-DNA in serum by molecular hybridisation. *J. Med. Virol.*, **9**, 273.